# Endovascular aneurysm repair in a centenarian: case report and systematic literature review

**DOI:** 10.1093/jscr/rjaa025

**Published:** 2020-04-03

**Authors:** Zara Sheikh, Stephen Crockett, Sadasivam Selvakumar

**Affiliations:** Department of Vascular Surgery, East and North Hertfordshire NHS Foundation Trust, Lister Hospital, Stevenage, UK

**Keywords:** Aged, 80 and over Aortic Aneurysm, Abdominal/surgery Endovascular Procedures

## Abstract

The prevalence of abdominal aortic aneurysms (AAA) in the nonagenarian and centenarian populations is set to increase. Endovascular aneurysm repair (EVAR) has been shown to be achievable with excellent outcomes in carefully selected nonagenarians. However, experience with centenarians is limited. We report the case of a 100-year-old who presented with a tender 8-cm AAA and successfully underwent EVAR. This report describes the second case of AAA repair in a centenarian in the literature and the first reported EVAR in this demographic. The patient survived for 2 years after the procedure, was free of EVAR or aneurysm-related complications. Furthermore, we present a systematic review of the existing literature and insights pertaining to outcomes in nonagenarians undergoing EVAR.

## INTRODUCTION

The introduction of endovascular aneurysm repair (EVAR) has extended the benefit of treatment of aneurysmal disease to those who would have previously been considered ‘unfit’ for open surgical repair. In 2018, there were 13 170 centenarians living in the UK, which has almost doubled since 2002 [1]. The volume of AAA work in the nonagenarian and centenarian population is anticipated to increase [2]. Several studies have suggested that EVAR procedures can be carried out with excellent technical success in nonagenarians; however, the centenarian population has not been specifically addressed [3–7]. At present, the only reported aneurysm repair in a centenarian is that of a 101-year-old undergoing open repair in the USA [8].

We report the case of a 100-year-old woman who successfully underwent emergency EVAR; she is currently the oldest patient in the literature to undergo EVAR. Furthermore, we present a systematic literature review of outcomes in nonagenarians following EVAR. This case raises important questions about how we assess survival advantage and make decisions in the centenarian population going forward.

## CASE REPORT

**Figure 1:**
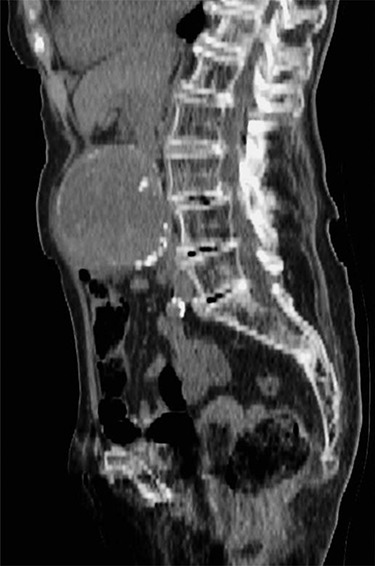
CT Aortogram demonstrating 8-cm infrarenal aneurysm.

A 100-year-old woman presented to the emergency department with a 4-day history of central abdominal pain. On examination, she was found to have a pulsatile, expansile aneurysm with stable observations. Computed tomography (CT) aortogram demonstrated an infrarenal AAA of 8 cm (Fig. 1). A detailed discussion took place with the patient and her family, explaining the high likelihood of mortality without surgery and the operative risks involved. The patient had a background of atrial fibrillation, hypertension, mild dementia and a recent hip replacement. She was able to walk for one mile and carry out most activities of daily living independently. Under these circumstances, we felt it appropriate to proceed, taking into consideration good preoperative functional status, limited comorbidities, anatomical suitability for EVAR under local anaesthetic and good recovery following a recent hemiarthroplasty. The patient underwent EVAR successfully, followed by an uneventful postoperative recovery. No endoleak or complication of the graft was noted at follow-up and the patient survived for 2 years following intervention.

**Figure 2:**
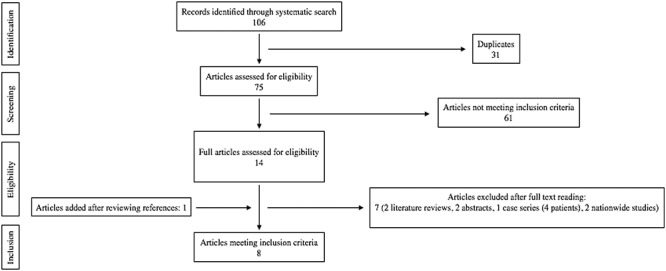
Systematic search strategy and article selection process.

## DISCUSSION

The future prevalence of AAAs in centenarians is likely to increase given the natural course of aneurysmal disease and increasing life expectancy. A systematic search of the Embase and Medline databases was conducted according to the PRISMA guidelines, using the terms nonagenarian and endovascular to identify single centre studies reporting EVAR outcomes in nonagenarians. This returned 106 results, 15 full text articles were reviewed after screening all titles and abstracts (Fig. 2). Eight articles met inclusion criteria (Table 1), evaluating the outcomes of EVAR in a total of 150 nonagenarian patients. Demographic data, aneurysm size, technical success, mean postoperative survival, perioperative morbidity and mortality were extracted from each study and weighted averages calculated. Mean age was 91.5 and 82.6% of those included were men. Mean aneurysm size was 6.8 cm and mean 30-day morbidity and mortality rates were 24.9% (7.4–40%) and 6.5%, respectively. Technical success was 96.7%. Two American nationwide studies were identified (Table 2) but excluded due to the possibility of duplication of results reported in the American single-centre studies. A limitation of the studies included was the lack of data on survival, comorbidities, and reasons for foregoing intervention in those not undergoing EVAR. This data would help to determine if there was any survival advantage of undergoing intervention. Furthermore, the mean age reported in all studies was in the low nineties and we cannot determine how many patients presenting in their late nineties were declined intervention.

**Table 1:** Single-centre studies reporting experiences with EVAR in nonagenarians

	*n*	Mean age (years)	Sex	Mean aneurysm size(cm)	Technical success (%)	Mean survival (months)	Percentage survival following surgery (year, %)	30-day complications (%)	30-day mortality (%)	Endoleaks
Lee *et al*. [3]	15	90.3	14 M	6.4	100	56	1. 91.7%	40	0	Type 1: 4
			1 F				2. 83.3%			Type 2: 9
							3. 71.4%			
							4. 57.1%			
							5. 38.1%			
Zhang *et al*. [4]	12	92.1	11 M 1 F	6.7	100	Mean survival of patients surviving perioperative period 28.5	1. 83.3	33	8.3	Type 1: 1
							3. 58.3			Type 2: 5
							5. 25.0			
Prenner *et al*. [9]	24	91.5	20 M	6.8	91.6	NR	1. 83.3	22	8.3	Type 1: 3 2 cases of indeterminate leak
			4 F				5. 19.3			
Goldstein *et al*. [5]	24	91.5	15 M	6.3	100	Mean survival of patients who had died at time of reporting 29.7	1. 83	33	4.2	Type 1: 1
			9 F			Mean survival of those still alive at time of reporting 36.1	2. 64			Type 2: 3
							3. 50			
Jim *et al*. [6]	18	91.2	12 M	6.8	100	Mean survival of patients surviving perioperative period who had died at time of reporting 17.5	1. 58.8	17	5.6	Type 1:1
			6 F			Mean survival of those still alive at time of reporting 25.6	2. 41.7			Type 2: 6
										Type 3: 1
Baril *et al*. [7]	18	92.4	18 M	7.3	100	Mean survival of patients who had died beyond 30 days: 34 months	NR	22	11	Type 1: 2
						Mean survival of those still alive at time of reporting: 17.4 months				Type 2: 2
Geisbusch *et al*. [10]	27	91.6	25 M 2 F	7.1	85	NR	1. 60.6	7.4	0	Type 1: 1
							3. 96.3			Type 2: 2
										Type 4: 3
Karnwal *et al*.	12	NR	9 M 3F	NR	NR	Mean survival in those surviving beyond 30 days 35.6	NR	NR	8	NR

**Table 2:** Nationwide studies evaluating outcomes of EVAR in nonagenarians

	*n*	Mean age (years)	Sex	Mean aneurysm size (cm)	Technical success (%)	Mean survival (months)	30-day complications (%)	30-day mortality (%)	Endoleaks
Hughes *et al*.	291	NR	218 M 73 F	NR	NR	NR	11.6	3.1	NR
Tsilimparis *et al*.	240	NR	NR	NR	NR	NR	16.2	9.1	NR

A single-centre study of 18 nonagenarians undergoing EVAR conducted by Jim *et al* reported a 100% technical success rate, with a 5.6% 30-day mortality and for those surviving beyond the first 30 days, a mean survival of 17.5 months [6]. Mortality rates were significant at 365 days and at 2 years, 41.2% and 58.3%, respectively, bringing into question the overall survival advantage conferred from the procedure. Similarly, Goldstein *et al.* [7] reviewed 24 nonagenarians undergoing EVAR, of which 8 were symptomatic at the time of repair, reporting a mean postoperative survival of 29.7 months in those who died during follow-up, and 36.1 months in patients still alive at the time of reporting [7]. Perioperative complication rates were higher in patients undergoing general anaesthetic, constituting five out of six systemic complications. There was one perioperative death and no deaths related to the aneurysm beyond 30 days. Comorbidities were assessed for each patient, complication rates significantly increased once >5 comorbidities were identified. Reintervention was required in one case for endoleak.

Undertaking any surgical intervention in patients of the nonagenarian and centenarian population requires careful consideration of comorbidities and functional status and perioperative involvement of a geriatrician should be considered. A retrospective study by Lee *et al.* looked at elective EVAR in 15 highly selected nonagenarians, with good premorbid status. They reported far more encouraging survival rates of 83.3% at 2 years, 71.4% at 3 years and 57% at 4 years, with a highly favourable mean survival of 56 months [3]. They identified an average of 2.7 comorbidities per patient in comparison to 3.5 or 4 comorbidities per patient in the studies conducted by Jim and Goldstein, which is likely to have contributed to differences in long-term survival. Reintervention rates were higher (27%) than the studies conducted by Jim and Goldstein (11% and 4.2%). Despite higher complication rates, Lee *et al.* [3] reported no 30-day mortalities and 100% rupture free survival extending to 5 years follow-up.

There is debate about the extent of survival advantage that could be conferred to the centenarian population and the ethics of operating on those of such advanced age. Few studies on life expectancy after the age of 100 have been conducted. Until further studies are conducted, evidence in this population relies on individual experience. Restoring a patient to their baseline to fulfil the average life expectancy of a centenarian without death related to aneurysm could be considered a successful outcome. This case proves that EVAR can be successfully achieved in carefully selected centenarians.
